# An S188V Mutation Alters Substrate Specificity of Non-Stereospecific α-Haloalkanoic Acid Dehalogenase E (DehE)

**DOI:** 10.1371/journal.pone.0121687

**Published:** 2015-03-27

**Authors:** Azzmer Azzar Abdul Hamid, Tengku Haziyamin Tengku Abdul Hamid, Roswanira Abdul Wahab, Mohd. Shahir Shamsir Omar, Fahrul Huyop

**Affiliations:** 1 Department of Biotechnology, Faculty of Science, IIUM, Bandar Indera Mahkota, 25200, Kuantan, Pahang, Malaysia; 2 Department of Chemistry, Faculty of Science, Universiti Teknologi Malaysia, 81310, UTM Johor Bahru, Johor, Malaysia; 3 Department of Biosciences and Health Sciences, Faculty of Biosciences and Medical Engineering, Universiti Teknologi Malaysia, 81310, UTM Johor Bahru, Johor, Malaysia; 4 Department of Biotechnology and Medical Engineering, Faculty of Biosciences and Medical Engineering, Universiti Teknologi Malaysia, 81310, UTM Johor Bahru, Johor, Malaysia; Russian Academy of Sciences, Institute for Biological Instrumentation, RUSSIAN FEDERATION

## Abstract

The non-stereospecific α-haloalkanoic acid dehalogenase E (DehE) degrades many halogenated compounds but is ineffective against β-halogenated compounds such as 3-chloropropionic acid (3CP). Using molecular dynamics (MD) simulations and site-directed mutagenesis we show here that introducing the mutation S188V into DehE improves substrate specificity towards 3CP. MD simulations showed that residues W34, F37, and S188 of DehE were crucial for substrate binding. DehE showed strong binding ability for D-2-chloropropionic acid (D-2CP) and L-2-chloropropionic acid (L-2CP) but less affinity for 3CP. This reduced affinity was attributed to weak hydrogen bonding between 3CP and residue S188, as the carboxylate of 3CP forms rapidly interconverting hydrogen bonds with the backbone amide and side chain hydroxyl group of S188. By replacing S188 with a valine residue, we reduced the inter-molecular distance and stabilised bonding of the carboxylate of 3CP to hydrogens of the substrate-binding residues. Therefore, the S188V can act on 3CP, although its affinity is less strong than for D-2CP and L-2CP as assessed by K_m_. This successful alteration of DehE substrate specificity may promote the application of protein engineering strategies to other dehalogenases, thereby generating valuable tools for future bioremediation technologies.

## Introduction

Haloalkanoic acids are used as active components in a wide range of herbicides, insecticides, plastics, and solvents. The broad application of these recalcitrant pollutants has contributed to environmental pollution and health problems. Certain enzymes, called dehalogenases, can degrade and detoxify haloalkanoic acids and other halogenated compounds and therefore represent potential solutions for this environmental problem.

Non-stereospecific α-haloalkanoic acid dehalogenase (E.C. 3.8.1.10) catalyses the hydrolytic dehalogenation of d- and l-2-haloalkanoic acids to yield l- and d-2-hydroxyalkanoic acids, respectively. This enzyme has been isolated and characterised from numerous microbial strains, including *Pseudomonas putida* PP3 [1,2], *Alcaligenes xylosoxidans* spp. *denitrificans* ABIV [3], *Pseudomonas* sp. 113 [4,5], and *Rhizobium* sp. RC1 [6]. The non-stereospecific α-haloalkanoic acid dehalogenase E (DehE) was isolated from *Rhizobium* sp. RC1 by growing this bacterium in medium containing 2,2-dichloropropionate as the sole source of carbon [7,8]. DehE was initially characterised along with the stereospecific dehalogenases, DehD and DehL, which were also isolated from *Rhizobium* sp. RC1 [6,9]. DehE catalyses the dehalogenation of many α-haloalkanoic acids but does not catalyse the dehalogenation of β-haloacids [6,10,11]. To date, only a few strains of bacteria have been shown to produce a dehalogenase that can remove a chloride ion from the β-carbon of 3-chloropropionic acid (3CP) [12–15]. Jing et al. [13] reported a dehalogenase from *Rhodococcus* sp. HJ1 that acts on 3-chlorobutyric and 2,2,3-trichlorobutyric acids, whereas Mesri et al. [14] isolated a haloacid dehalogenase from a bacterial strain that uses 3CP as its sole source of carbon. In addition, Hamid et al. [16] characterised the ability of β-haloalkanoic acid dehalogenase from *Pseudomonas* sp. B6P to degrade 3CP. Recently, computational tools were used to characterise DehE, and it was shown to contain a single active-site cavity within the monomeric enzyme [17]. The DehE active site permits the binding of 3CP, as well as the α-chlorinated acid compounds d-2CP and l-2CP, at residues W34, F37, and S188.

Mutagenesis studies by our group have confirmed that the DehE residues W34, F37, and S188 serve as the substrate-binding residues for d-2CP and l-2CP and that they are necessary for catalysis [18]. The dehalogenation reaction facilitated by DehE is similar to those catalysed by other non-stereospecific haloalkanoic acid dehalogenases [5,19]. The enzymatic reaction utilises a direct attack mechanism that is triggered when D189 activates a water molecule to generate a hydroxide ion. This ion consequently attacks the C_2_ atom of the substrate to liberate the halide ion. Interactions between α-haloalkanoic acid compounds and DehE have recently been described [17], but there are no published reports concerning interactions between DehE and β-haloalkanoic acid. In fact, studies concerning non-stereospecific haloalkanoic acid dehalogenase are rare.

To better characterise the catalytic activity of DehE—specifically its substrate specificity with respect to β-haloacids—it is important to determine the molecular mechanisms that prevent its catalysis of 3CP. Briefly, enzyme engineering can be conceptually divided into three categories, namely activity engineering (changing k_cat_), substrate specificity engineering, and stability engineering (improving resistance to pH, temperature, etc.) [20]. In the current study we focused on engineering the substrate specificity of DehE by varying spatial interactions, structural stability, and orientation of the DehE-3CP complex to more closely resemble the DehE-d-2CP and DehE-l-2CP complexes. This could improve our understanding of the multifaceted interaction between DehE and 3CP.

Nakamura et al. [21] investigated the catalytic interactions of l-specific haloalkanoic acid dehalogenase using a Molecular dynamics (MD) approach. In contrast, Schmidberger et al. [2] used docking simulation to elucidate binding interactions associated with DehI from *Pseudomonas putida* PP3. Here, our goal was to improve the substrate specificity of DehE towards 3CP by characterising substrate-docking interactions within DehE-complexes. We used MD simulations and site-directed mutagenesis analysis. DehE is very specific for 2CP, in this paper we rationally design the enzyme in order to allow it to react with a non activated chloroacid namely 3CP. This study may promote the application of protein-engineering strategies to α-haloalkanoic acid dehalogenases to generate improved reagents for bioremediation technologies.

## Materials and Methods

### Data set

The DehE structure used in this study was obtained from the Swiss-Model web server [22] as described [17]. The structures of d-2CP, l-2CP and 3CP were used as small molecules for the investigation. These structures were obtained from PubChem, a database maintained by the National Center for Biotechnology Information [23]. Each complex between DehE and d-2CP, l-2CP, or 3CP was generated using Autodock Tools version 4.2, as described [17]. Site-specific mutation of S188 was performed using PyMOL [24], and all complexes including S188V-3CP mutant were simulated using GROMACS version 4.5.1 [25].

### Molecular dynamics simulations

Each of the three complexes involving d-2CP, l-2CP, or 3CP docked to DehE was used as the starting point for MD simulation. The Gromacs package 4.5.1 adopting the GROMOS 53a6 force field parameter was utilised. Structures were solvated in a cubic box, using periodic boundary conditions and the simple point charge water model at a density of 0.998 g/cm^3^. The dimension of the box was setup with the box edge approximately 10 Å from the molecule (s) periphery. The water molecules were allowed to relax using the position-restraint procedure. The PRODRG server [26] was used to generate ligand topologies, and 8 Na^+^ counter ions were added to neutralise the total charge of the system. Energy minimisation of enzyme-substrate complexes was performed using 800 steps of steepest descent. Following this procedure the system was equilibrated at constant temperature (303 K) and pressure (1 atm) for 50 ps. Finally, the equilibrated structure was subjected to production stage whereby NPT ensemble was used for over 10,000 ps with an integration time step of 2 fs. The non-bonded list was generated using an atom-based cut-off of 10 Å. Long-range electrostatic interactions were handled using the particle-mesh Ewald algorithm [27]. All bond lengths involving hydrogen atoms were constrained using the Lincs algorithm [28]. Trajectory snapshots were collected every 4 ps. Root mean square deviations (RMSDs), root mean square of fluctuations (RMSFs), total energy, and the distance between atoms were analysed using Gromacs utilities (g_rms, g_energy and g_dist).

### Data analysis

The RMSD plot was used to determine the equilibrated structure, whereas RMSF was used to identify local flexibility of the system from the MD trajectory. The 10,000 ps RMSD and RMSF plots were constructed for DehE complexes (DehE-d-2CP, DehE-l-2CP, and DehE-3CP), and equilibrated structures of the complexes were extracted at 8,000 ps for the substrate-binding conformation. Total energy plots were generated to determine the energy of DehE-substrate complexes. At 4,000–4,500 ps, which are the significant range of distance fluctuations for DehE-3CP complex, the structures were extracted to analyse the atomic movement and substrate orientation.

### Molecular visualisation

Three-dimensional structures of the complexes were visualised using PyMOL [24] and Visual Molecular Dynamics [29]. Distance variations between substrate-binding residues and substrates were monitored every frame during the course of the simulation.

### Site-directed mutagenesis of DehE

Overlapping PCR was used to introduce site-specific mutations. The primers were 5'-ctatcaccatggccccgcagtc(agc)gatttccaagctc-3' and 5'-gagcttggaaatcgac(gct)tgcggggccatggtgatag-3'. Base changes in the relevant triplets are shown in bold and the triplets are underlined. The original triplets are given in the parentheses. Each PCR reaction (50 μl) contained 50 ng *pET-22b(+)-dehE*, 5 μl 10× reaction buffer, 1.5 μl dNTPs, 1.25 μl forward primer (125 ng), 1.5 μl reverse primer (125 ng), and 2.5 U *Pfu* ultra High-Fidelity DNA polymerase. The thermal cycle program was 95°C for 5 min, followed by 18 cycles of 95°C for 50 s, 55°C for 50 s, and 68°C for 7 min. A final extension step of 68°C for 7 min was included. The PCR product was treated with *Dpn*I for 1 h and transformed into *Escherichia coli* XL1-Blue competent cells. Wild-type and mutant *dehE* were expressed as described [18]. Bacteria were sonicated, and cell-free extracts containing dehalogenase were harvested for enzymatic assays as previously described [6].

### Dehalogenase activity assay

The preparation of cell free extracts (CFE) and the measurement of dehalogenase activity were made as previously described [10]. Dehalogenase activity for the kinetic studies were performed in triplicates by measuring the amount of chloride ions released from 4.7 ml distilled water containing 0.1 M Tris-acetate (pH 7.6) and 50 μl of 0.1 M 3CP or D-2 chloropropionic acid (D-2CP) or L-2 chloropropionic acid (L-2CP) as the substrate at 30°C. After 5 min of equilibration at 30°C, the reaction was initiated by adding soluble cell-free extracts or pure dehalogenase enzyme to a final volume of 5 ml. Samples (1.0 ml) were removed at 5-min intervals. The released chloride ion in the assay mixture was measured using the colourimetric technique of Bergmann and Sanik [30] on a UV/VIS spectrophotometer (Perkin-Elmer). Colour was allowed to develop for 10 min at room temperature and measured at A_460nm_. Control experiment, lacking enzyme preparation and/or denatured enzyme was included in each set of assay to detect spontaneous halogen released. Unit of enzyme activity (U) was defined as the amount of enzyme that catalysed the release of 1 μmol chloride/min. Specific activity (1 μmol Cl^–^·min^–1^·mg^–1^) was calculated as the unit of enzyme activity per mg of total protein. Protein was determined by the biuret procedure with crystalline egg albumin as a standard [31]. Enzyme specific activity is defined as the μmole of chloride liberated per milligram protein in 10 min under the stated conditions.

### Analytical methods

**Fig 1:**
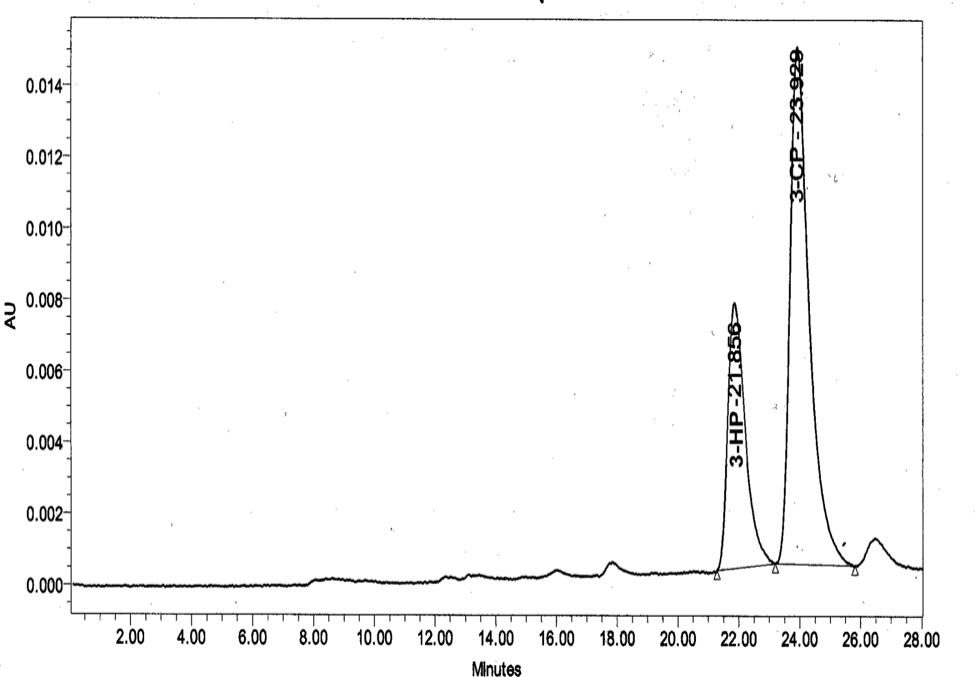
HPLC profile of 3HP (t_R_: 21.85) and 3CP (t_R_: 23.92) standards (10 mM each).

High-performance liquid chromatography (HPLC) analysis was performed using Waters 2690 Alliance equipped with separations module with waters 996 Photodiode Array Detector and Hi-Plex H 300 mm x 7.7 mm column (Agilent Technologies, USA). An Isocratic mobile phase of 0.005 N H_2_SO_4_ with flow rate of 0.6 ml/min was used. Detection was carried out at 210 nm at 40°C. Under this condition, the retention time (t_R_) of 3-hydroxypropionic acid (3HP) and 3CP were 21.85 and 23.92, respectively (Fig. 1).

### Protein purification

The purification of dehalogenase protein from cell free extracts of recombinant *E*.*coli* strains was achieved using the method described by Huyop et al. [10].

## Results

### MD simulation of DehE-d-2CP, DehE-l-2CP, and DehE-3CP

**Fig 2:**
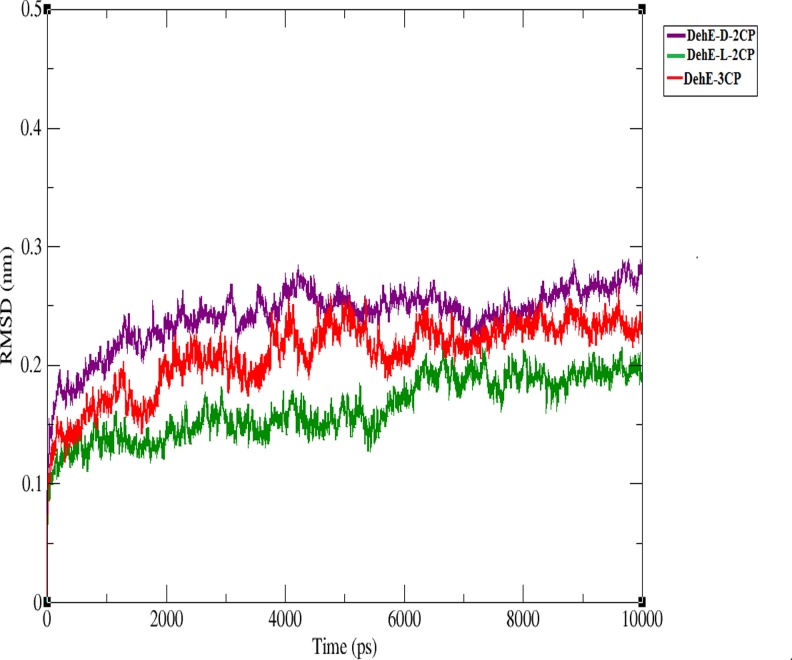
Root mean square deviations (RMSDs) of the alpha-carbon positions during 10,000-ps simulations.

**Fig 3:**
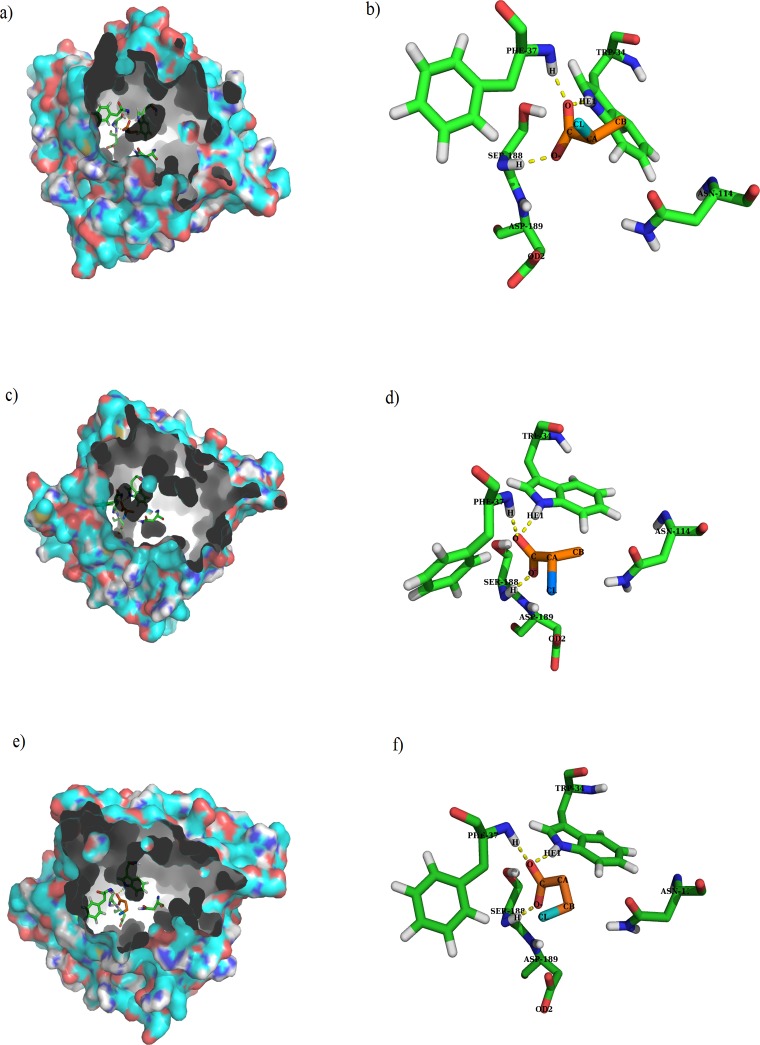
Molecular visualisation of substrate binding. (A) d-2CP in the binding pocket of DehE. (B) Hydrogen bonding between d-2CP and W34, F37, and S188 in the DehE-d-2CP complex. (C) l-2CP in binding pocket of DehE. (D) Hydrogen bonding between l-2CP and W34, F37, and S188 in the DehE-l-2CP complex. (E) 3CP in the binding pocket of DehE. (F) Hydrogen bonding between 3CP and W34, F37, and S188 in the DehE-3CP complex.

MD simulations were performed for the DehE-d-2CP, DehE-l-2CP, and DehE-3CP complexes. Conformational stability during MD simulations was represented by the RMSD of the alpha-carbon atoms and three-dimensional structures of DehE-d-2CP, DehE-l-2CP, and DehE-3CP complexes were simulated in explicit water molecules throughout the 10,000 ps of MD calculations. Water molecules are pertinent for actual simulation of DehE catalysed hydrolysis of the 2-haloalkanoic acid substrates since such reaction requires presence of water. Fig. 2 shows the structures reached a stable equilibrium during the simulation. The RMSD for DehE-d-2CP was steady at ~2.5 Å after 4,000 ps, whereas DehE-l-2CP was steady at ~1.7 Å after 7,000 ps. Overall, the RMSD for both complexes remained fairly constant throughout the 10,000 ps of MD simulation, suggesting that the simulation time was sufficient to obtain equilibrated forms of the DehE-substrate complexes. The RMSD of the DehE-3CP complex showed trends that were similar to DehE-d-2CP and DehE-l-2CP. The DehE-3CP complex, however, recorded a low RMSD value and plateaued at **~**2.2 Å after 4,000 ps which implied attainment of a stable DehE-3CP complex. Therefore, it was appropriate that the equilibrated structures for all complexes were extracted at 8,000 ps. Confirmation of the substrate binding residues is established by observing formation of new hydrogen bonds between the enzyme and substrate. These analyses revealed that the carboxylate group of d- and l-2CP was strongly hydrogen bonded to residues W34 and the backbone amides of F37, and S188 (Fig. 3). Similar hydrogen bonding interactions were observed for the DehE-3CP complex which corroborate a previous report demonstrating that DehE interacts and binds to 3CP through residues W34, F37, and S188 [17]. It can be inferred that these residues were vital for substrate bonding due to their location within the hydrogen bonding distance (< 3 Å) to the substrate moiety.

**Fig 4:**
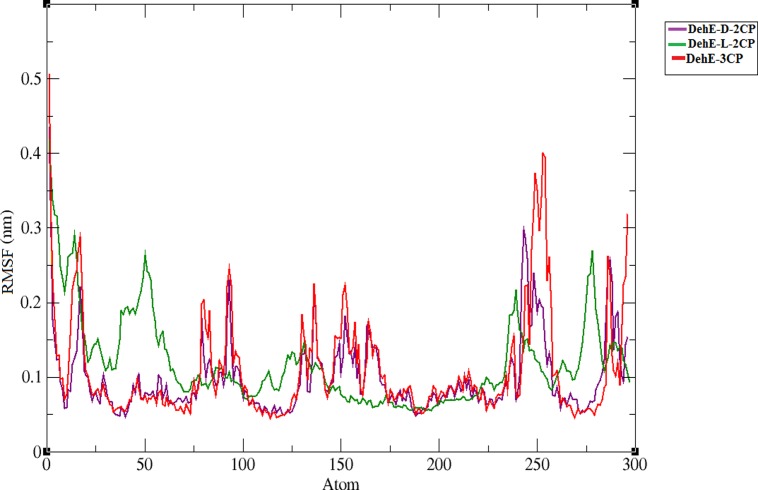
Root mean square fluctuations (RMSFs) of the alpha-carbon positions of DehE complexes.

**Fig 5:**
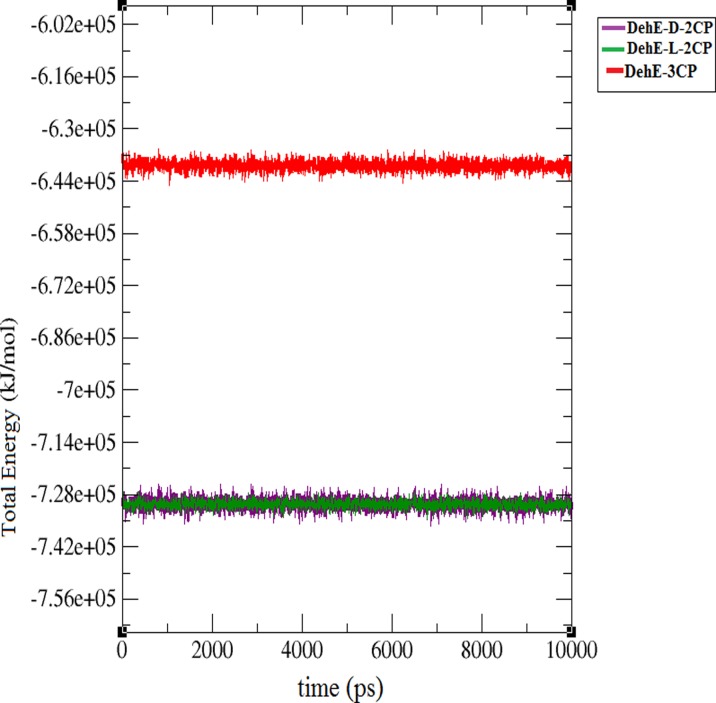
Total energy of DehE complexes during 10,000-ps simulations.

The RMSF of each backbone atom within each DehE complex was calculated over the course of the simulation to assess local flexibility of the system. All DehE complexes showed a large fluctuation of the N-terminal residue. Residues exhibited dynamic properties within these complex structures (Fig. 4), with all DehE-3CP and DehE-d-2CP residues fluctuating in a similar manner. In contrast, residues within the DehE-l-2CP complex fluctuated slightly differently (except for the first and last 75 residues). DehE-l-2CP had a more rigid and compact structure, whereas DehE-d-2CP and DehE-3CP exhibited similar flexibility. Calculating the total energy of each molecule shed further light on DehE binding preferences. From the first picosecond of the MD simulation, total energy for each of these complexes was stable (Fig. 5), suggesting high levels of structural stability. DehE-3CP had a higher total energy (–6.40* 10^5^ kJ/mol) than DehE-d-2CP or DehE-l-2CP, with a difference of ~ 1.00 kJ/mol.

### Atomic distance analysis of DehE-d-2CP, DehE-l-2CP, and DehE-3CP

**Fig 6:**
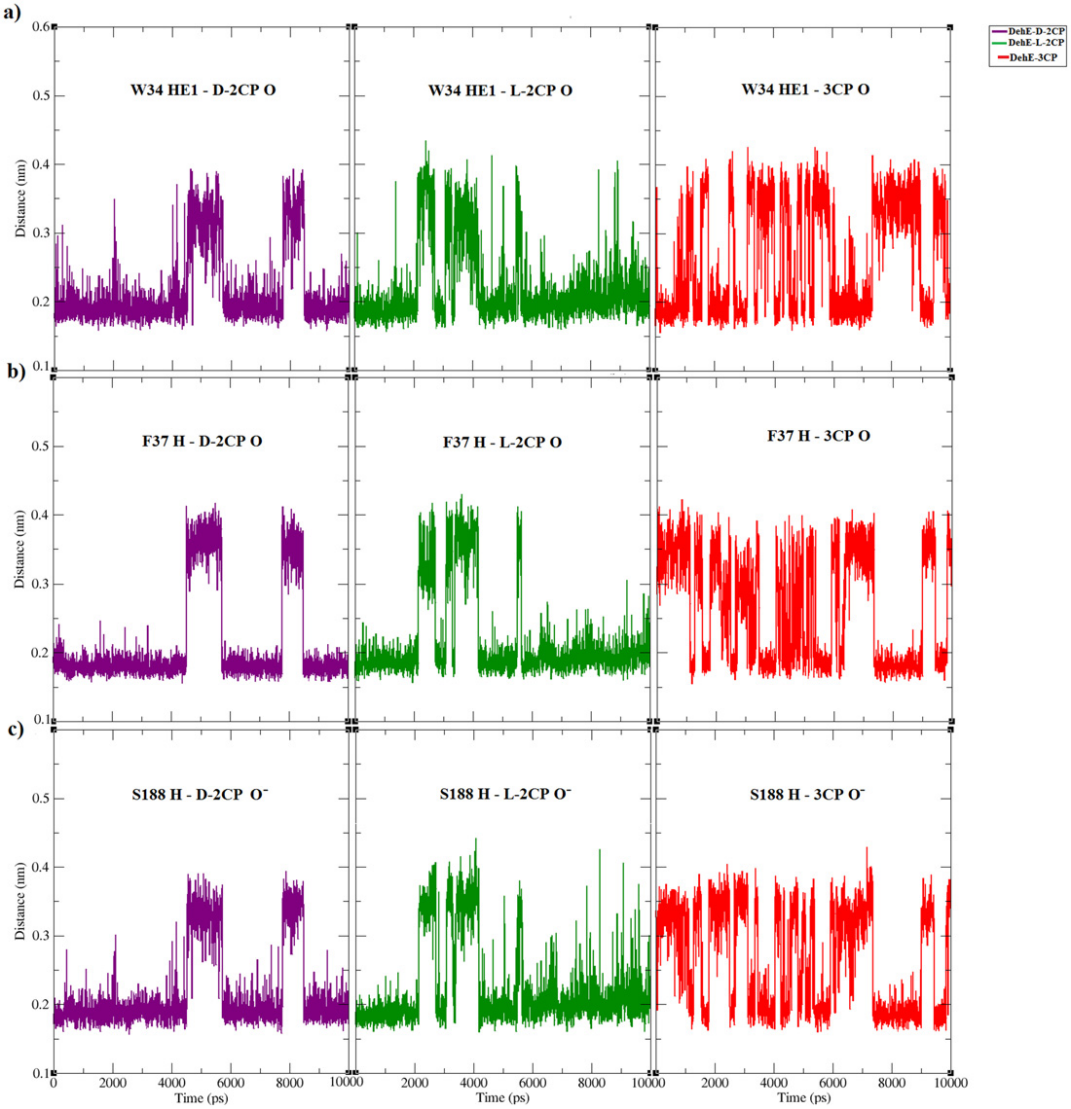
Atomic distance analysis of MD trajectories associated with each of the DehE-d-2CP, DehE-l-2CP, and DehE-3CP complexes. (A) Time course of distances between HE1 of W34 and O of d-2CP (purple line), HE1 of W34 and O of l-2CP (green line), and HE1 of W34 and O of 3CP (red line). (B) Time course of distances between H of F37 and O of d-2CP (purple line), H of F37 and O of l-2CP (green line), and H of F37 and O of 3CP (red line). (C) Time course of distances between H of S188 and O^-^ of d-2CP (purple line), H of S188 and O^-^ of l-2CP (green line), and H of S188 and O^-^ of 3CP O^-^ (red line).

Active-site residues of DehE were previously verified using site-directed mutagenesis, revealing that W34, F37, and S188 are crucial for substrate binding [18]. Here we measured distances between these binding residues and substrate in DehE complexes. Calculation of binding distances throughout simulation time (0 to 10,000 ps) is important to assess the enzyme-substrate affinity level of DehE towards 3CP. Nakamura et al. [21] reported that residue K151 of L-DEX exhibited high affinity towards 2-chloropropionic acid as exemplified by the small atomic distance (< 3 Å) throughout simulation time. In this study, distances between the carboxylate oxygen (O) of d-2CP or l-2CP and HE1 of W34 (the H atom of the amino acid side chain) remained fairly constant at ~1.8 Å. Stability of these atomic distances substantiate previous studies concerning the role W34 plays in binding d-2CP or l-2CP [17]. Similar observations were made for F37 and S188. Distances between the carboxylate oxygen (O) of d-2CP or l-2CP and the hydrogen atom of the backbone amide for F37 was ~1.8 Å, as was the distance between carboxylate oxygen of d-2CP or l-2CP and the hydrogen atom of the backbone amide for S188 (Fig. 6).

Evaluation of DehE-3CP revealed that the atomic distance between O of 3CP and HE1 of W34 fluctuated between 1.8 and 4.0 Å. This instability indicates a weak intermolecular interaction, which generally leads to unstable substrate binding and sub-optimal substrate orientation, consequently, the inability to carry out catalysis. Residues, F37 and S188 also displayed similar atomic distance fluctuations when bound to 3CP. The atomic distance between O of 3CP and H of F37 varied between 1.8 and 4.0 Å, whereas the distance between O^-^ of 3CP and H of S188 ranged between 1.8 and 3.8 Å (Fig. 6).

**Fig 7:**
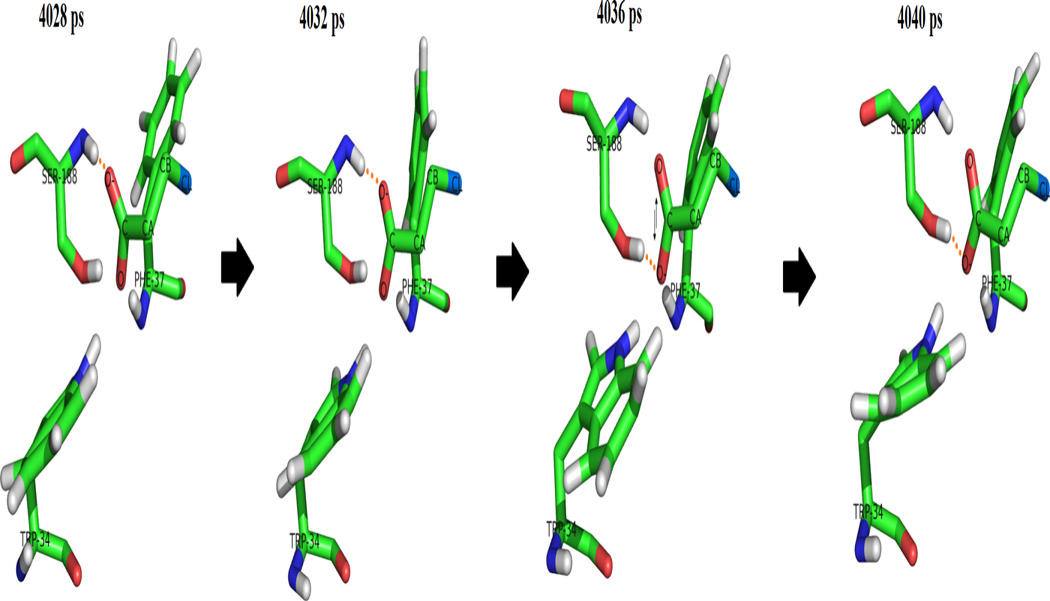
Interconversion of hydrogen bonds between the 3CP carboxylate and S188 in DehE-3CP complex.

For DehE-3CP, significant fluctuations in binding distances were observed between 2,000 and 6,000 ps which were caused by the improper orientation of the 3CP carboxylate within the active site. As seen in Fig. 7, the carboxylate O^-^ of the 3CP formed a hydrogen bond with the hydrogen (H) of the amide group of the S188 backbone from 4,028 ps of the simulation. However, every 8 ps thereafter the O^-^ of the carboxylate twisted, relocated, and formed a hydrogen bond with the hydrogen (H) of the hydroxyl group of the S188 side chain. This repeated hydrogen bond relocation was continuously observed during 126 sampled frames. As a result, a stable DehE-3CP complex is not attained, and catalysis does not occur. These data support to the argument that DehE does not dehalogenate 3CP because 3CP carboxylate cannot form a strong hydrogen bond with S188.

### Stabilisation of the DehE-3CP complex

**Fig 8:**
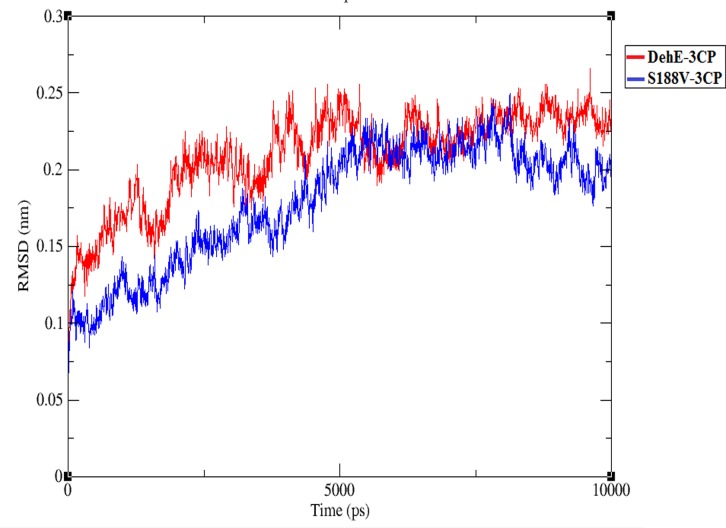
Root mean square deviations (RMSDs) of the alpha-carbon positions for S188V-3CP and DehE-3CP during 10,000-ps simulations.

**Fig 9:**
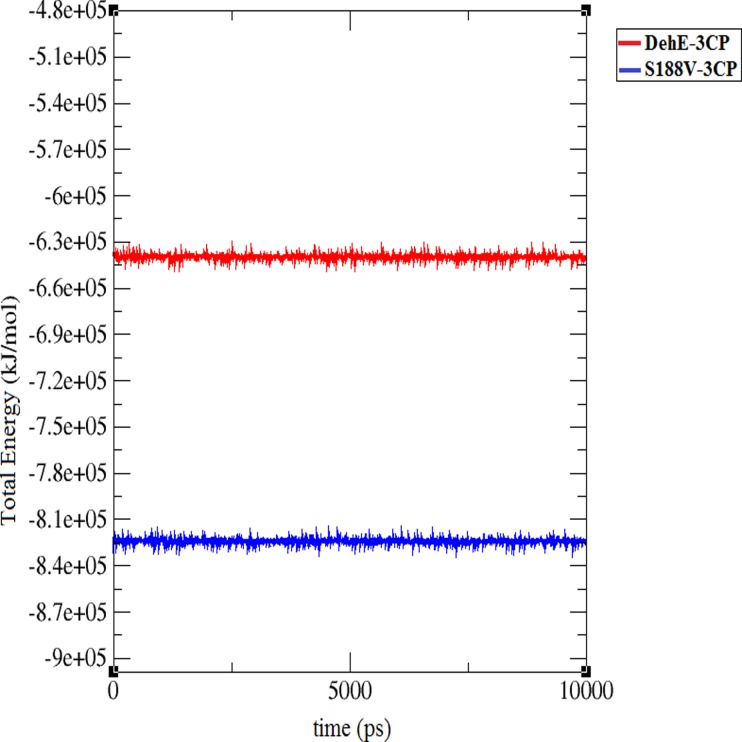
The total energies of S188V-3CP and DehE-3CP during 10,000-ps simulations.

At the beginning of the trajectory (t = 0 ps) until ~5,500 ps, RMSD of the S188V-3CP complex was lower (1.0–2.0 Å) than for DehE-3CP (1.25–2.5 Å) (Fig. 8). Deviation values for both complexes rapidly increased during the initial 5,500 ps, indicating dynamic structural changes during the equilibration process. Between 5,500 and 7,500 ps, the RMSDs of S188V-3CP and DehE-3CP were stable at ~2.0 Å. After 7,500 ps, however, the deviation value for S188V-3CP began to decrease, whereas the DehE-3CP RMSD remained at ~2.25 Å. The difference in deviation values between the complexes after 7,500 ps could be attributed to structural changes associated with the S188V mutation. Concerning energy plots, S188V-3CP possessed a low total energy of approximately—8.4* 10^5^ kJ/mol (Fig. 9). As such, this mutation significantly reduced the average total energy of S188–3CP compared with other DehE complexes. This likely resulted from the stabilisation of bonds between the carboxylate group of 3CP and the backbone of the valine residue within the DehE active site.

**Fig 10:**
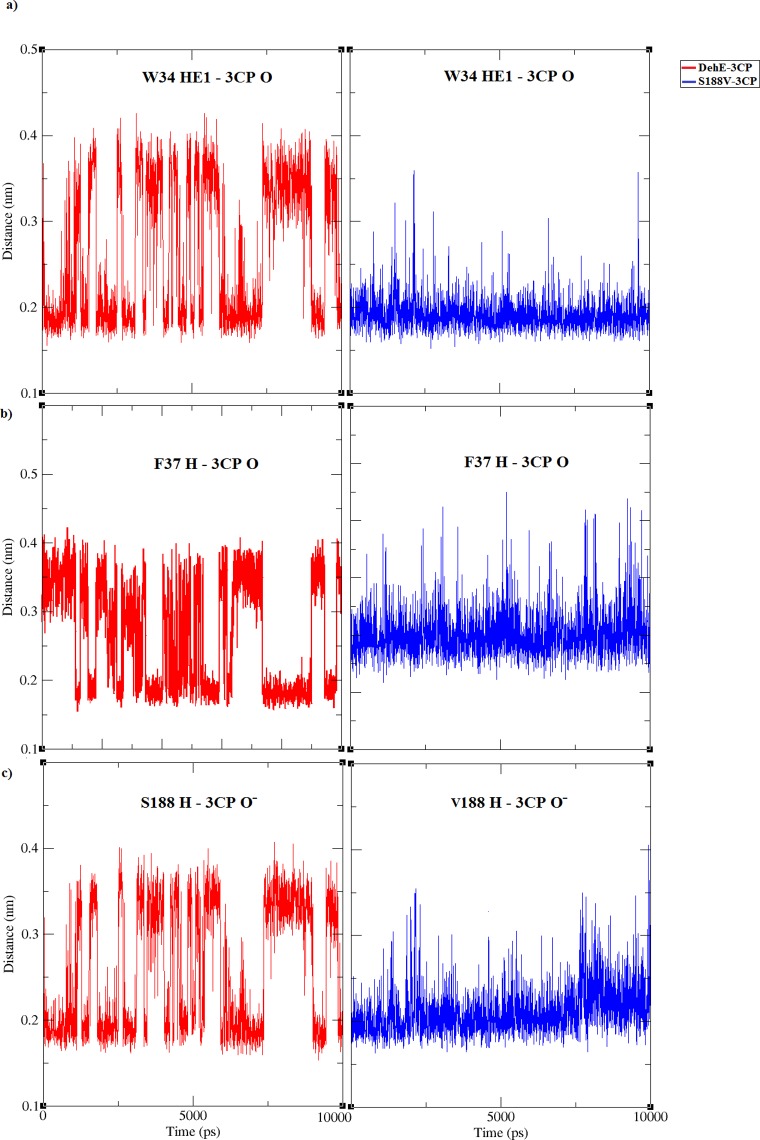
Atomic distance analysis of the MD trajectory of DehE-3CP and S188V-3CP complexes. (A) Time courses of the distance between HE1 of W34 and O of 3CP for DehE-3CP (red line) and HE1 of W34 and O of 3CP for S188V-3CP (blue line). (B) Time courses of the distance between H of F37 and O of 3CP for DehE-3CP (red line) and of H of F37 and O of 3CP for S188V-3CP (blue line). (C) Time courses of the distance between H of S188 and O^-^of 3CP for DehE-3CP (red line) and of H of V188 and O^-^of 3CP for S188V-3CP (blue line)

Atomic distance analysis revealed that the distance between the carboxylate O of 3CP and HE1 of W34, H of F37, or H of S188V was maintained at ~2.0 Å until the end of the simulation (Fig. 10). Binding distances for S188V-3CP did not fluctuate, suggesting that the S188V mutation strengthened the hydrogen bond between DehE and 3CP, thereby generating a more rigid DehE-3CP complex. The S188V-3CP complex equilibrated more quickly than DehE-3CP, exhibited less structural fluctuations, and was more stable in water.

### Dehalogenase activity of S188V in cell-free extracts

Dehalogenase activity was assessed by measuring the release of chloride ions from 3CP after exposure to enzyme-containing cell-free extracts. For extracts containing wild-type DehE, no dehalogenase activity was detected, as expected. For extracts containing the S188V mutant, however, activity towards 3CP was detected with a specific activity of 2.79 ± 0.01 μmol Cl^–^·min^-1^·mg^-1^. Controls without adding any cell free extracts prepared from wildtype DehE and S188V DehE mutant did not show any chloride release in the reaction mixture containing 3CP as substrate. Cell free extract from S188V DehE mutant was heat denatured at 70°C for 15 min and used in the similar reaction did not show any chloride released from 3CP suggesting enzyme from S188V DehE mutant can solely act on the 3CP substrate. Summary of enzyme specific activity for 3CP compared to the activity for D- and L-CP for both wild type and mutant was described in Table 1.

**Table 1:** Specific activity of enzyme in cell free extract prepared from wild type DehE and S188V mutant DehE.

	Specific activity (μmol Cl^–^·min^-1^·mg^-1^)		
**Substrate**	**3CP**	**D-2CP**	**L-2CP**
Wild type DehE	No activity	4.80 ± 0.02	5.50 ± 0.04
S188V DehE mutant	2.79 ± 0.01	3.79 ± 0.03	4.21 ± 0.02

### HPLC analysis

**Fig 11:**
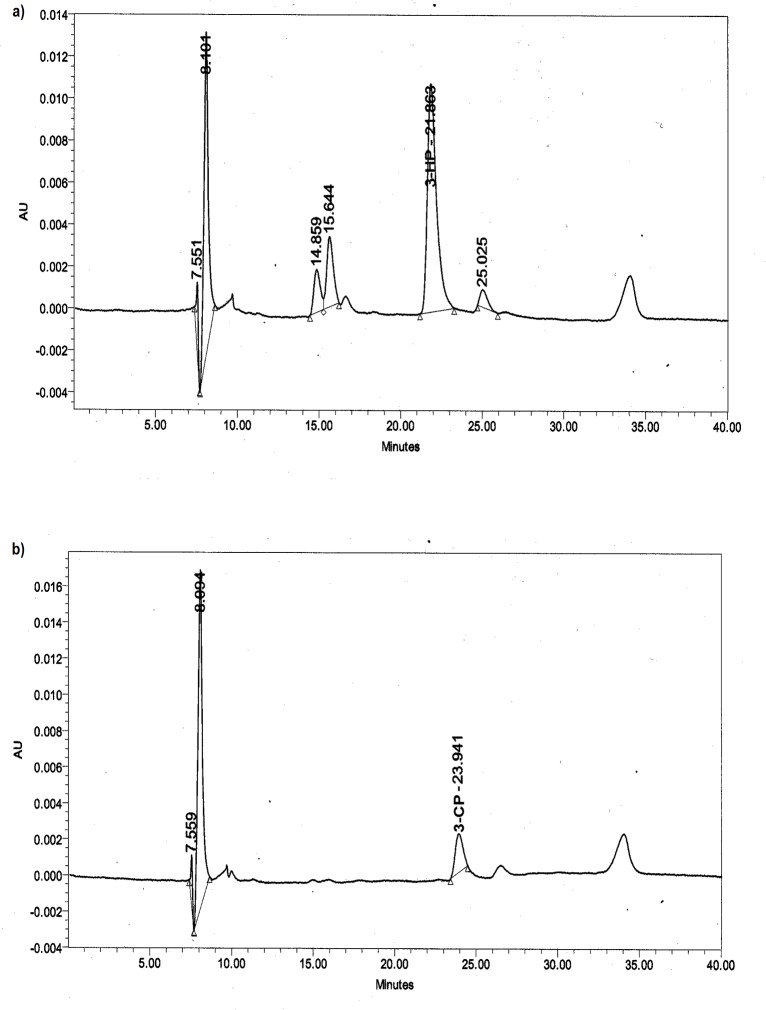
HPLC profile of 3CP degradation. (a): degradation of 3CP producing 3HP (t_R_ 21.86). (b) The 3CP (t_R_: 23.94) was not degraded by the enzyme in cell free extract from the wild type DehE.

The dehalogenation product of 3CP from the enzyme assay reaction using both extracts from wild type and mutant were evaluated using HPLC. The enzyme assay was allowed to run for 30 min. The enzyme assay reaction using extract from S188V DehE mutant gave rise to 7 peaks (Fig. 11a). The peak at 21.86 shared similar elution time with 3HP standard, whereas an assay using extract from wild type DehE did not show a peak at 21.86 suggesting there is no degradation of 3CP (Fig. 11b). Using enzyme from wild type DehE, the assay was left for more than one hour and no traces of 3HP was detected in HPLC suggesting there is no enzyme activity occurs in the assay reaction mixture of wild type DehE.

### Purification of cell free extracts and kinetic analyses

**Fig 12:**
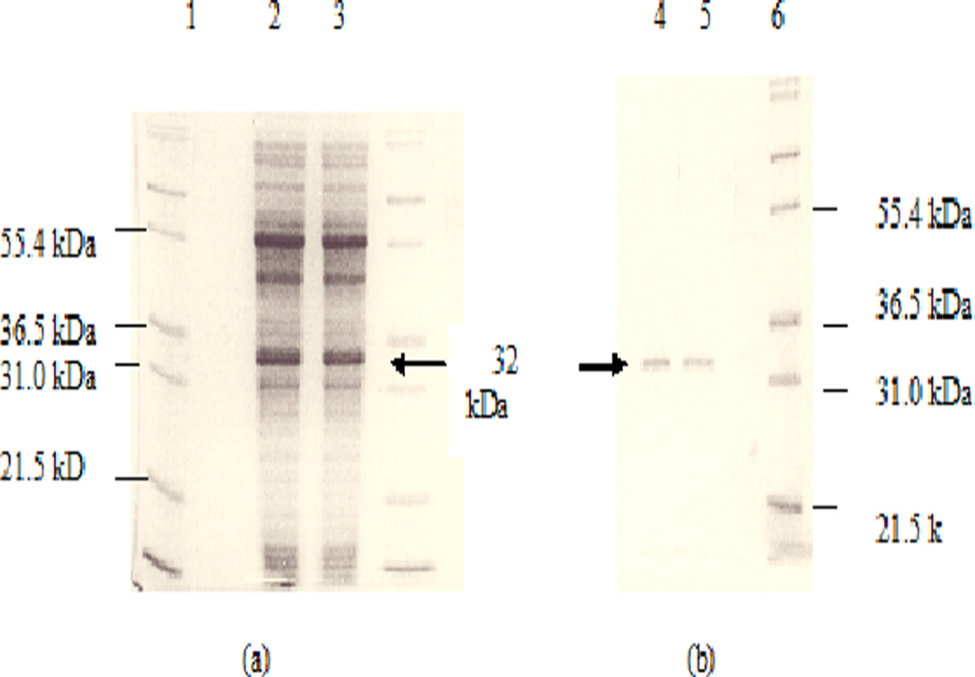
SDS-PAGE analysis of cell free extracts and pure proteins. a) Lane 1: Protein markers (Invitrogen); Lane 2: cell free extract of wild type DehE; Lane 3: S188V DehE mutant. b) Lane 4: pure enzyme extract of wild type DehE (32 kDa) Lane 5: pure enzyme extract of S188V DehE mutant (32 kDa); Lane 6: Protein markers (Invitrogen).

Cell free extracts of wild type DehE and S188V DehE mutant were purified. The fractions had 2.2 U and 2.5 U enzyme and a specific acivity of 1.9 U/mg and 2.5 U/mg with D,L2-chloropropionic acid (D,L2CP) as substrate, respectively with a recovery of at least 90% in total. Analysis of the fraction by SDS-PAGE showed that at least 95% of the protein seen was accounted for by a 32 kDa band (Fig. 12). Current analysis was sufficiently pure for further characterisation. Characterisation by apparent kinetic analyses of pure extract from mutant and wild type dehalogenases was performed using 3CP, D-2CP and L-2CP substrates.

Results of the kinetic experiments are shown in Table 2. It was found that S188V mutant DehE can act on all substrates and does not show any substrate specificity. However, the K_m_ results did show significant differences between D-2CP, L-2CP and 3CP with lower K_m_ values for L-2CP compared to D-2CP. The calculated k_cat_ values were varied, with D-2CP being the fastest substrate molecule converted to product, whereas L-2CP was the slowest. The catalytic efficiencies for the various substrates by DehE mutant showed 3CP has higher specificity constant value than D-2CP.

**Table 2:** Apparent kinetic parameters for hydrolysis of chloropropionate substrates by wild type and mutant enzymes determined under standard conditions.

Source of purified enzyme	Substrates	K_cat_ (s^-1^)	K_m_ (M)	Specificity constant M^-1^sec^-1^
Wild type DehE	3CP	Not available	Not available	Not available
	D-2CP	7.76	(4.8 ± 0.03) x 10^-4^	1.61 x 10 ^4^
	L-2CP	12.32	(4.3 ± 0.02) x 10^-4^	2.86 x 10 ^4^
S188V DehE mutant	3CP	12.55	(4.9 ± 0.02) x 10^-4^	2.56 x 10 ^4^
	D-2CP	9.18	(4.5 ± 0.01) x 10^-4^	2.04 x 10 ^4^
	L-2CP	13.11	(4.2 ± 0.02) x 10^-4^	3.12 x 10 ^4^

## Discussion

Calculation of the RMSD in a MD simulation serves to assess the stability of the complex structures as a whole. The absence of asymptotic behaviour in the DehE-3CP complex indicated that the complex had indeed formed and that structural unfolding did not occur. DehE-3CP showed similar structural changes as DehE-d-2CP, whereas DehE-l-2CP exhibited only slight fluctuations and equilibrated much later (after 7,000 ps). Because DehE-l-2CP deviated less and had lower RMSD values than complexes involving d-2CP and 3CP, l-2CP likely had stronger interactions with residues W34, F37, and S188, resulting in a more stable complex. These results explain why DehE catalyses l-2CP more efficiently than d-2CP (Table 2). It has also been reported that DehE has a smaller K_m_ value and a higher specificity constant for l-2CP than for d-2CP [10]. However, comparing structural changes between these two complexes and DehE-3CP does not sufficiently explain the preference that DehE exhibits towards d- and l-2CP over 3CP. Analyses of RMSF values and estimations of total energies revealed that the DehE-3CP complex is more flexible and has higher energy than the other complexes. We attribute these properties to unstable substrate binding due to weak hydrogen bonds and inappropriate substrate orientations within the active site. This may explain why DehE cannot degrade 3CP. Hydrogen bonds formed between polar atoms are generally < 3.0 Å [32] and maintain correct substrate orientation within the active site as seen in trajectories of MD analyses. Meanwhile, the calculated total energies critically reflect the possible attainment of a catalytically competent or destabilized conformation of the DehE-3CP complex. With regards to the binding affinity, it is accepted that combination between low energy of the enzyme-substrate complex and a short binding distance portray high affinity of the enzyme to the substrate.

Theoretically, a single molecule that contains two electron-rich moieties should position these moieties as far apart as possible to minimise repulsion and reduce the overall energy of the molecule. With this principle in mind, the distance between the large β-chloride ion and the negatively charged carboxylate should be maximised within the 3CP molecule. It is possible that this distance exceeds the available finite space within the DehE active site, thereby reducing binding affinity. The binding step is very important during enzyme catalysis as it lowers the free energy barrier of the substrate, which expedites the forward reaction [32,33]. The 3CP molecule, therefore, may be too large to form stable hydrogen bonds with DehE. In addition, Parisini et al. [34] described a complex halogen bonding feature involving σ-hole charge distribution allowing halogen atom to act as an electron donor. Such bonding between small halogen-substituted ligands and protein receptor can facilitate and expedite enzymatic dehalogenations.

Polar residues such as serine are associated with local amino acid flexibility and amino acid disorder [35]. This instability is thought to impart structural specificity [36]. In the case of DehE-3CP, however, the combination of an expanded 3CP substrate and molecular flexibility near S188 likely impedes substrate binding. To improve substrate binding in this context, we sought to stabilise residues surrounding S188 by replacing the S188 side chain with one that could not participate in hydrogen bonding [37] and that did not interfere with the overall protein structure. The non-polar valine was chosen to replace S188 because of its comparable molecular weight, its compatibility with hydrophobic protein interiors [35,38] and is generally well tolerated [39]. The S188V mutant reduced the intermolecular distance from ~2.5 Å (DehE-3CP) to ~2.0 Å (S188V-3CP). A more rigid S188V-3CP structure likely translated into a well-oriented 3CP molecule within the DehE active site.

According to the hydrophobicity scale of Kyte and Doolittle, substituting the weakly hydrophobic serine (relative hydrophobicity = –0.80) with a more hydrophobic valine (4.62) tends to increase the internal hydrophobicity of the active site, favouring the binding of hydrophobic substrates [40,41] and promote catalysis [40,42]. Importantly, introduction of the valine residue eliminated hydrogen bonding between the carboxylate of 3CP and the side-chain moiety. Bonds were only formed with the hydrogen of the main-chain amide of valine, thereby eliminating the erratic hydrogen bond interconversion seen with the DehE-3CP complex. Consequently, DehE substrate specificity and affinity were shifted towards the non-natural substrate 3CP. It was reported that substrate specificity of D-glucose dehydrogenase isoenzyme of *Bacillus megaterium* IAM 1030 was successfully altered following substitution with more bulky and hydrophobic amino acid residues. Of the six mutants created, the G259A variant exhibited the narrowest substrate specificity, whilst retaining comparable catalytic activity and thermostability to the wild-type enzyme [43]. Most importantly, by increasing the catalytic repertoire of DehE, the enzyme can degrade a wider range of halogenated acid compounds, potentially providing a ‘one pot’ solution for the successful removal of halogenated compounds from the environment.

**Fig 13:**
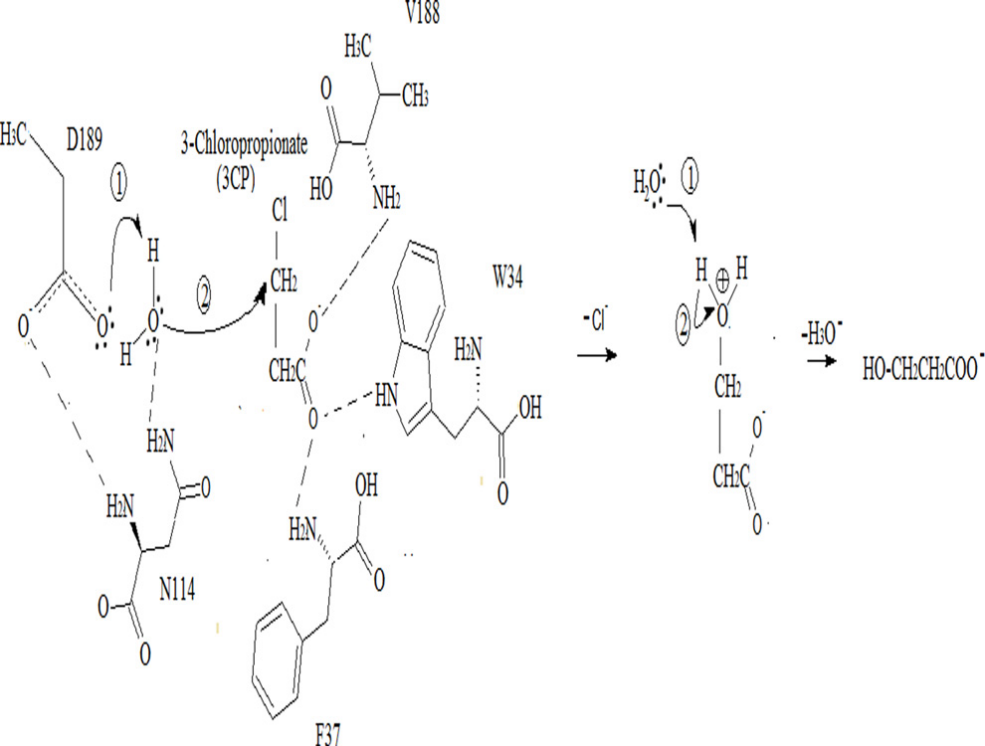
A possible reaction mechanism of hydrolysis catalysed by DehE mutant. Asp189 incorporated with Asn 114 both act as base at the DehE enzyme structure. This initiate water activation. The nucleophilic attack of the carbon-halogen bond of the substrate takes place and resulted in formation of intermediate. Finally, the release of halide (chloride ion) from the compound and formation of hydroxylated product can be detected.

Mutation of S188 to valine modified the substrate specificity of DehE and allowed it to dehalogenate chlorinated propanoic acid in the β-position. An enzymatic assay showed that the mutant DehE degraded 3CP, similar to a proposed pathway by Hughes [44] for 3-hydroxypropionate (3HP) production by hydrolytic dechlorination of 3CP. This observation correlated with the higher catalytic efficiencies (specificity constant) value in Table 2 for 3CP. However, the kinetic values for D- and L-2CP remained somewhat alike for the mutated protein suggesting possible occurrence of similar enzyme-substrate interaction when subjected to MD simulation. A possible reaction catalysed by DehE mutant is illustrated in Fig. 13. Degradation of β-substituted halogenated aliphatic acid has also been reported in *Pseudomonas* sp. B6P [14]. The 3HP can be obtained from substrate like glycerol [45,46]. However, the current study implies that 3HP can also be produced from 3CP by dehalogenation process.

Although stability of the enzyme-substrate complex is critical for biocatalysis, other important criteria should not be ignored. For example, the presence of water and the orientation of the catalytic residue are also important for hydrolytic dehalogenation. In this study, we found that weak substrate bonding associated with 3CP caused instability and prevented degradation. We confirmed this hypothesis using site-directed mutagenesis. We also calculated distances and angles between catalytic residues and water molecules for each of the complexes DehE-d-2CP, DehE-l-2CP, and DehE-3CP. We found that both the distance and angle between D189 and H of a water molecule remained constant (~1.8Å and 80°) throughout the 10,000-ps, simulation, suggesting that the enzyme is stable (unpublished data) and that degradation is not prevented by the orientation of the catalytic residue relative to water. Similarly, catalytic orientation has also been discussed regarding the hydrolytic mechanism of L-specific haloalkanoic acid dehalogenase, L-DEX YL [21].

## Conclusions

Advances in computational enzymology have made computational methods more reliable and yielded more accurate results when studying enzymatic reactions. We demonstrated that DehE does not catalyse the degradation of 3CP because a strong bond is not formed between these two molecules and thus a stable DehE-3CP complex is not achieved. Weak hydrogen bonding between DehE and 3CP was evidenced by atomic distance fluctuations between the two molecules. MD simulations and site-directed mutagenesis revealed that substituting S188 with the small hydrophobic residue valine made the DehE-3CP complex more rigid and elevated affinity for 3CP. The neutral valine side chain neutralised the erratic interconversion of hydrogen bonding associated with the carboxylate of serine at position 188 and also restricted bonding to the hydrogen of the main-chain amide in DehE-3CP. Based on our findings, we believe it is possible to engineer a more versatile DehE through directed mutations within the active site. In this way, the enzyme-substrate complex can be further stabilised to enhance substrate specificity and direct affinity towards non-degradable substrates.
